# Association between quality of the diet and cardiometabolic risk factors in postmenopausal women

**DOI:** 10.1186/1475-2891-13-121

**Published:** 2014-12-22

**Authors:** Danyelle de Almeida Ventura, Vânia de Matos Fonseca, Eloane Gonçalves Ramos, Lizanka Paola Figueiredo Marinheiro, Rita Adriana Gomes de Souza, Celia Regina Moutinho de Miranda Chaves, Maria Virginia Marques Peixoto

**Affiliations:** Instituto Fernandes Figueira (IFF/Fiocruz), Avenida Rui Barbosa, 716-Flamengo, Rio de Janeiro, RJ Brazil; University of Mato Grosso, Av. Fernando Corrêa da Costa, n° 2367-Bairro Boa Esperança, Cuiabá, Mato Grosso Brazil

**Keywords:** Aging, Menopause, Eating habits

## Abstract

**Background:**

Climateric is a phase of women’s life marked by the transition from the reproductive to the non-reproductive period. In addition to overall weight gain, the menopause is also associated with the increase of abdominal fat. We used The Healthy Eating Index as a summary measure to evaluate the major components and the quality of women’s diet after the onset of the menopause. This study aims at examining the association between the quality of the diet and cardiometabolic risk factors in postmenopausal women.

**Methods:**

Cross-sectional study including 215 postmenopausal women attending a public outpatient clinic. The 24-hour dietary recall method was used to assess the food intake and to establish the Healthy Eating Index. Diets were then classified as appropriate diet (>80 points), diet “requiring improvement” (80–51 points), and poor diet (<51 points). Cardiometabolic risk factors included abdominal obesity, dyslipidemia, diabetes mellitus, and hypertension. The Fisher’s exact test was utilized for the Statistical analysis.

**Results:**

The analysis of the food intake showed that the average daily intake of lipids (36.7%) and sodium (2829.9 mg) were above the recommended. Only 8.8% of the women performed moderate or intense physical exercises on a regular basis. The diet was considered poor in 16.3%, “requiring improvement” in 82.8%, and appropriate for only 0.9% of the women. The study detected increased waist circumference in 92.1% of the participants. The mean concentration of triglycerides was of 183.3 mg/dl, and 130.7 mg/dl for cholesterol (Low Density Lipoprotein).

**Conclusion:**

Women consume a low quality diet, possibly due to the low intake of vegetables and fruits and excessive consumption of sodium. These inappropriate eating habits are associated with and, have a negative impact on the cardiometabolic risk factors such as abdominal obesity.

## Background

The World Health Organization (WHO) defines climateric as a biological phase and not a pathological process, a significant period of female aging, characterized by the establishment of a physiological progressive state of hypoestrogenism, which ends with the permanent cessation of the menstrual cycles. Menopause is a mark of this phase and is acknowledged only one year after the occurrence of the last menstrual cycle [1]. In addition to an overall increase in body weight, menopause has been associated with a greater accumulation of abdominal fat [2]. This unfavorable change in the body fat distribution contributes to explain the increased cardiovascular risk during this stage of life [3].

The association among nutrients, food, and non-communicable diseases (NCD) can be studied with the same tools used to assess individual food consumption, such as the 24-hour recall, which reflects the previous day of the individual food intake. The Healthy Eating Index (HEI) developed using data from the 24-hour dietary recall, is a summary measure of the main components of an individual’s diet. It facilitates the assessment of a diet’s quality, of either populations or groups of individuals. Without reducing the assessment of a single component alone, it takes into consideration the complexity of a given diet, and allows for an indirect assessment of nutrients [4].

Climateric is a topic of concern in women’s health, not only for the uncomfortable symptoms it engenders, but also for its potential impact on public health. The latter includes the high prevalence of non-communicable diseases due to a new food pattern of the Brazilian population, the increased consumption of processed foods with high levels of saturated fat, sugar, and salt, in parallel with a growing elderly population [5].

Within this context, the primary purpose of this study is to verify the association between diet’s quality and cardiometabolic risk factors in postmenopausal women as well as to contribute to the validation of findings in different living conditions and geographic settings.

## Methods

A cross-sectional observational study consisting of a convenience sample of 234 postmenopausal women undergoing treatment at the gynecology outpatient clinic of Instituto Fernandes Figueira (IFF), within the endocrinology and urogynecology subspecialties, from October 2011 to October 2012. The inclusion criteria were: women aged ≥ 45 years and without a menstrual period for 12 consecutive months or more. Exclusion criteria were: dietary counseling with physician or nutritionist, uncontrolled thyroid disease, special diet or vegetarian and extremely low dietary intake (<500 kcal/day) or extremely high (>4000 kcal/day). Two hundred and fifteen women met the criteria and underwent all the required exams. The Research Ethics Committee of IFF approved this study and each participant signed a Statement of Informed Consent.

Nutritional status was estimated by The Body Mass Index (BMI = weight/height^2^). Weight and height were measured with a standard beam balance scales (Filizola®, Brazil) and with a stadiometer (Wiso®, Brazil), respectively [6, 7]. The nutritional status was interpreted according to WHO recommendations. Women (<65 years) were classified as normal weight (18.5–24.9Kg/m^2^), as overweight (25–29.9Kg/m^2^), or obese (≥30Kg/m^2^). Elderly women (≥65 years) as lean (<22Kg/m^2^), normal weight (22–27Kg/m^2^), or overweight (>27Kg/m^2^) [8].

Waist circumference (WC) was measured at the midpoint between the iliac crest and the lower border of the last floating rib at the end of a normal expiration, using inelastic tape. Each measurement was duplicated and the mean value recorded. Abdominal fat was estimated indirectly by the measurement of the waist circumference, and rated high when waist >80 cm [8].

We used the cutoff point of the Brazilian Society of Cardiology for adults, with isolated systolic hypertension ≥ 140 × 90 mmHg to classify blood pressure level [9].

Blood was collected from each subject after 12-hour fasting. These were used to determine Triglycerides (TG), total cholesterol (TC), high-density lipoprotein cholesterol (HDLC) and glucose levels. The low-density lipoprotein cholesterol (LDLC) was calculated using Friedewald et al.’s formula: LDLC = Total cholesterol - (triglycerides/5 + HDLC) [10]. Normal values were: TC <200 mg/dl, HDLC > 50 mg/dl, LDLC <100 mg/dl, TG <150 mg/dl and glucose levels <100 mg/dl [11].

Regarding lifestyle, women were classified as active (self-evaluation) or sedentary. Furthermore, this study adopted the recommendation of the Centers for Disease Control and Prevention (2008) that defines as active women those who undergo moderate or intense aerobic activity for 30 minutes at least five times a week or muscle-strengthening activities two or more days per week [12].

Regarding food intake we used the acceptable macronutrient Distribution Ranges proposed in 2005 by the National Academy of Sciences being 45%–65% for carbohydrates, 10%–35% for proteins, and 20–35% for lipids of a standard total daily intake of 2000Kcal [13]. According to the Feeding Guidelines for the Brazilian Population, the daily intake of saturated fat, cholesterol, and sodium should be less than, 10%, 300 mg and 2400 mg respectively. In addition, the Ministry of Health recommends in the Feeding Guidelines for the Brazilian Population, a minimum daily intake of 3 servings of fruits (each serving of 70 kcal) and 4 servings of greeneries and vegetables (each serving of 15 kcal) [14].

Dietary intake data were obtained through dietary recall of each participant, with reported information on food intake over a 24-hour period preceding the interview or more often the previous day [15]. The nutritional values of food products, consumed and registered in the 24-hour dietary recall, were analysed using the Nutrition Support Programme (Nutwin, [16]). Food products that were not in the database were introduced, using the Brazilian Food Composition Tables (TACO, [17]) and Pinheiro et al. [18]. The data on food intake were used to calculate the HEI scores. The HEI comprises ten components being five food groups (cereal, breads, roots and tubers; fruits and vegetables; milk and dairy products; meat, eggs and beans), four nutrients (saturated fat, total fat, cholesterol, and sodium), plus a measure of dietary variety. Each of ten components contributes with 10 points to the maximum possible score of 100 [19].

The servings of the food follow the daily recommendation of the Feeding Guide for the Brazilian Population as per 1000 Kcal. Based on the total energy calculated by adding up all items of a given food group, we calculated the number of consumed portions of this group, based on the energy content of a defined portion [20]. Each component of the HEI had a score from zero to ten, and intermediate values were recorded as a proportion of the consumption by group. Thus, ten points, would be scored if the consumption in the group of cereals, breads, tubers and roots was of 450/1000 kcal. Likewise for the group of greenery and vegetables, 22.5/1000 kcal and the fruits group 105/1000 kcal. The same for the consumption of milk and dairy products 180/1000 kcal and the meat, eggs and beans 122.5/1000 kcal. The calculation of all other components followed the same rational being total fat <30%; saturated fat <10%; cholesterol < 300 mg/day, sodium ≤ 2,4 mg/day, and the variety in the diet characterized by the consumption of 8 or more different types of food daily [21]. According to the final score, the consumption was classified as “poor” diet (HEI <51 points), diet “requiring improvement” (HEI between 51 and 80 points), and healthy diet (HEI > 80 points) [19].

The data was stored with double entry on access database and processed by a software developed in R language and the statistical analysis performed with the Statistical Package for Social Sciences software – SPSS, version 13. The continuous variables depicted by measures of central tendency (mean or median), scatter plot, and of minimum and maximum values. For the comparison of categorical variables, the Fisher’s exact test or they Chi-squared test were used with α = 0.05 for statistical significance and 95% confidence interval.

## Results and discussion

Table 1 shows the main characteristics of the study group. Median age was 58.9 years and median age at menopause 49.0 years. Regarding the pattern of food consumption, the results of this study are analogous to those reported by Tardivo et al. [21] on the association between diet quality and metabolic risk indicators in postmenopausal women in the state of São Paulo. The median HEI scores was 60.0 and the total daily calorie intake averaged 1607.8 Kcal showed by these authors were close to our findings, respectively 60.7 for HEI scores and 1619.4 Kcal (Table 1). The participants of this study consumed a slightly higher percentage of protein (median 17.3 versus 15.4%), and carbohydrates (49.9 versus 46.0%), but a lower consumption of lipids (35.6 versus 38.3%). Most women surveyed consumed either a diet “requiring improvement” (82.8%) or poor (16.3%), and only 0.9% ate an adequate diet. Tardivo et al. also showed a similar pattern regarding poor diet and requiring improvement (48.5%), with an adequate diet recorded for 3% of women [21].

**Table 1:** **Clinical characteristics, laboratory values, dietary intakes and Healthy Eating Index score**

Characteristics	Average	Median (P25; P75)*	Range**
***Sociodemographic***			
Age (years)	59.3	58.9 (53.4; 64.6)	44.5–90.1
Education (years)	5.85	8 (4.0; 8.0)	0–8
N° of people living in the house	2.88	3 (2.0; 4.0)	1–8
Per capita income (R$)	1678.5	1240 (850; 2000)	300–12000
***Outpatient clinics***			
Age at Menopause (years)	47.2	49 (44; 51)	30–60
Length of menopause (years)	12.0	10.7 (5.5;17.2)	0.8–34.3
BMI (Kg/m^2^)	28.3	28.0 (24.6;31.6)	18.1–42.5
WC (cm)	95.4	94.7 (87.3; 103)	65–134
Diastolic blood pressure (mmHg)	81.5	80 (70; 90)	60–140
Systolic blood pressure (mmHg)	131.2	130 (120; 140)	90–260
***Lifestyle***			
Exercises leisure (minutes/week)	256.1	225 (120; 300)	20–900
***Inflammatory markers***			
Glucose levels (mg/dL)	111.9	103 (94; 117)	66–469
TC (mg/dl)	222.3	218 (196; 250)	91–336
LDLC (mg/dl)	130.7	128 (106; 154)	63–246
HDLC (mg/dl)	55.7	55 (46; 64)	29–100
TG (mg/dl)	183.3	165 (120; 220)	60–558
***Food Intake***			
Total energy value (kcal/d)	1737.8	1619.4 (1286.1; 2041.2)	698.8–3955.3
Protein (%)	18.2	17.3 (14.2; 21.6)	8.1–44.5
Carbohydrate (%)	48.8	49.9 (41.5; 56.0)	14.6–73.6
Lipids (%)	36.7	35.6 (30.2; 41.7)	16.6–68.2
Saturated Fat (%)	10.3	9.8 (7.8; 12.7)	3.1–25.2
Cholesterol (mg)	230.5	178.2 (122.8; 282.3)	14.7–989.8
Sodium (mg)	2829.9	2521.5 (1843.5; 3603,8)	530.5–10596.2
HEI (score)	60.3	60.7 (54.2; 66.9)	33.0–84.2

*Data are expressed as median with 25th and 75th percentiles in parentheses.

**Values minimum–maximum.

The vast majority (94.9%) reported a protein intake within the recommended limits and it did not account significantly to the quality of the diet (Table 2) as opposed to Tardivo’s study where all macronutrients contributed significantly to determining the quality of the diet [21]. Regarding women who consumed a poor diet our results are comparable to their findings as to higher consumption of calories, lipids, saturated fat, cholesterol and sodium, as well as carbohydrates below the recommended intake (Table 2).

**Table 2:** **Association between Healthy Eating Index scores and clinical characteristics, inflammatory markers and dietary intake**

Characteristics	N Total = 215	p-value ^a^	Poor diet ^b^ n = 35 (16.3%)	Diet needs improvement ^c^ n = 178 (82.8%)	Good diet ^d^ n = 2 (0.9%)
***Outpatient clinics***					
Hormone therapy		0.57			
Yes	30 (14.0%)		3 (10.0%)	27 (90.0%)	0 (0%)
No	185 (86.0%)		32 (17.3%)	151 (81.6%)	2 (1.1%)
BMI <65 years old		0.45			
Eutrophic	48 (28.7%)		5 (10.4%)	43 (89.6%)	0 (0%)
Overweight	63 (37.7%)		13 (20.6%)	49 (77.8%)	1 (1.6%)
Obese	56 (33.6%)		11 (19.6%)	44 (78.6%)	1 (1.8%)
BMI ≥ 65 years old		0.15			
Lean	5 (10.4%)		0 (0%)	5 (100.0%)	0 (0%)
Eutrophic	14 (29.2%)		0 (0%)	14 (100.0%)	0 (0%)
Overweight	29 (60.4%)		6 (20.7%)	23 (79.3%)	0 (0%)
WC (cm)		0.78			
<80	17 (7.9%)		2 (11.8%)	15 (88.2%)	0 (0%)
≥80	198 (92.1%)		33 (16.7%)	163 (82.3%)	2 (1.0%)
High blood pressure		0.38			
Yes	68 (31.6%)		14 (20.6%)	54 (79.4%)	0 (0%)
No	147 (68.4%)		21 (14.3%)	124 (84.4%)	2 (1.4%)
Self-reported lifestyle		0.09			
Active	71 (33.0%)		9 (12.7%)	60 (84.5%)	2 (2.8%)
Sedentary	144 (67.0%)		26 (18.1%)	118 (81.9%)	0 (0%)
Aerobic exercises		0.81			
Yes	14 (6.5%)		2 (14.3%)	11 (78.6%)	1 (7.1%)
No	25 (11.6%)		2 (8.0%)	22 (88.0%)	1 (4.0%)
Strengthening exercises		0.58			
Yes	5 (2.3%)		1 (20.0%)	4 (80.0%)	0 (0%)
No	34 (15.8%)		3 (8.8%)	29 (85.3%)	2 (5.9%)
***Inflammatory markers***					
Glucose levels (mg/dL)		0.73			
<100	80 (37.2%)		14 (17.5%)	66 (82.5%)	0 (0%)
≥100	135 (62.8%)		21 (15.6%)	112 (83.0%)	2 (1.5%)
TC (mg/dl)		0.27			
<200	60 (27.9%)		13 (21.7%)	47 (78.3%)	0 (0%)
≥200	155 (72.1%)		22 (14.2%)	131 (84.5%)	2 (1.3%)
LDLC (mg/dl)		0.75			
<100	40 (18.6%)		5 (12.5%)	35 (87.5%)	0 (0%)
≥100	175 (81.4%)		30 (17.1%)	143 (81.7%)	2 (1.1%)
HDLC (mg/dl)		0.37			
<50	75 (34.9%)		15 (20.0%)	60 (80.0%)	0 (0%)
≥50	140 (65.1%)		20 (14.3%)	118 (84.3%)	2 (1.4%)
TG (mg/dl)		0.58			
<150	88 (40.9%)		13 (14.8%)	75 (85.2%)	0 (0%)
≥150	127 (59.1%)		22 (17.3%)	103 (81.1%)	2 (1.6%)
***Food intake***					
Total energy intake (kcal/day)		**<0.001**			
<2000	160 (74.4%)		10 (6.3%)	148 (92.5%)	2 (1.3%)
≥2000	55 (25.6%)		25 (45.5%)	30 (54.5%)	0 (0.0%)
Protein (%)		0.21			
<10	7 (3.3%)		0 (0.0%)	7 (100.0%)	0 (0.0%)
10–35	204 (94.9%)		33 (16.2%)	169 (82.8%)	2 (1.0%)
>35	4 (1.9%)		2 (50.0%)	2 (50.0%)	0 (0.0%)
Carbohydrate (%)		**0.001**			
<45	77 (35.8%)		20 (26.0%)	57 (74.0%)	0 (0.0%)
45–65	129 (60.0%)		15 (11.6%)	112 (86.8%)	2 (1.6%)
>65	9 (4.2%)		0 (0.0%)	9 (100.0%)	0 (0.0%)
Lipids %		**0.001**			
<20	7 (3.3%)		0 (0.0%)	7 (100.0%)	0 (0.0%)
15–30	94 (43.7%)		4 (4.3%)	88 (93.6%)	2 (2.1%)
>30	114 (53.0%)		31 (27.2%)	83 (72.8%)	0 (0.0%)
Saturated fat (%)		**<0.001**			
<10	111 (51.6%)		9 (8.1%)	100 (90.1%)	2 (1.8%)
≥10	104 (48.4%)		26 (25.0%)	78 (75.0%)	0 (0.0%)
Cholesterol (mg)		**<0.001**			
≤300	168 (78.1%)		14 (8.3%)	152 (90.5%)	2 (1.2%)
>300	47 (21.9%)		21 (44.7%)	26 (55.3%)	0 (0.0%)
Sodium (mg)		**<0.001**			
≤2400	96 (44.7%)		4 (4.2%)	91 (94.8%)	1 (1.0%)
>2400	119 (55.3%)		31 (26.1%)	87 (73.1%)	1 (0.8%)
Vegetables and greenery intake		0.1			
≥4 servings	23 (10.7%)		5 (21.7%)	17 (73.9%)	1 (4.3%)
<4 servings	192 (89.3%)		30 (15.6%)	161 (83.9%)	1 (0.5%)
Fruit Intake		1			
≥3 servings	2 (0.9%)		0 (0.0%)	2 (100.0%)	0 (0.0%)
<3 servings	213 (99.1%)		35 (16.4%)	176 (82.6%)	2 (0.9%)

Data are expressed in numbers and percentage between parentheses.

^a^statistical difference between groups p <0.05 (Fisher’s Exact Test).

^b^HEI < 51 points.

^c^HEI between 51 and 80 points.

^d^HEI > 80 points.

When evaluating the mean quality scores separately for each component of the HEI, the worst records relate to the fruits and greeneries and, the vegetables groups (Table 3). The group of meat, eggs and beans stood out as the best score, possibly linked to the daily consumption of beans, a typical and vital component of the Brazilian diet. These findings are in agreement with the results of the Household Budget Survey (POF 2008–2009), which showed a profile of food consumption that combines traditional Brazilian diet of rice and beans with foods that are high in calories with low content of nutrients and, well below the recommended intake for fruits, greeneries and vegetables [22].

**Table 3:** **Mean, median, minimum and maximum score of the Healthy Eating Index components**

HEI components	Mean	Median	Score Min	Score Max
Cereal, bread, tubers, roots	3.96	3.77	0.52 (0.5)	9.8 (0.5)
Vegetables and greenery	2.13	0.00	0 (67.4)	10 (10.7)
Fruits	1.83	1.39	0 (1.9)	10 (0.9)
Milk and dairy	4.87	4.52	0 (9.8)	10 (17.2)
Meat, eggs and legumes	10.0	10.0	9.0 (0.5)	10 (99.5)
Total fat	5.57	6.20	0 (19.5)	10 (24.7)
Saturated fat	7.29	10.0	0 (10.2)	10 (51.6)
Cholesterol	8.47	10.0	0 (11.6)	10 (78.1)
Sodium	7.33	9.49	0 (7.9)	10 (44.7)
Variety of diet	8.90	10	2.0 (1.9)	10 (65.6)
Diet quality index	60.3	60.7	33.0 (0.5)	84.2 (0.5)

Data are expressed as number and percentage in parentheses.

Levy Costa et al. reported an evolving change of food availability, distribution and food intake patterns at the household and population levels since the 70’s, leading to the current profile. Their findings are consistent with the rising prevalence of overweight and obesity and the increasing rate of NCDs and their contribution to the morbidity and mortality profiles of the population. This diet configuration, called “western” or “westernized” includes a high intake of salt and sugar, a reduced consumption of fruits, fiber and vegetables and an increased level of total and saturated fats [23].

Pereira et al. compared the food consumption of women aged 35 or more in two population based cross sectional studies undertook in Rio de Janeiro, Brasil, in 1995–1996 (n = 1.014) e 2004–2005 (n = 1.001). The prevalence of obesity (BMI ≥ 30 kg/m2) raised from 16.6% para 24% in 10 years. Pereira et al. also show that changes in dietary intake of adult women in the Metropolitan Region of Rio de Janeiro are in disagreement with the recommendations for healthy eating. There was an increase in the consumption of processed foods with high-energy and there was a decrease in the consumption of fruits, milk, beans, roots, tubers and meats. In this population group, the shift from the traditional Brazilian food habits is detrimental to the overall quality of diets and possibly contributes to the occurrence of overweight and obesity. There is also an increased risk of developing metabolic disorders and other non-communicable chronic diseases [24]. Most women of this study had an inadequate consumption of fruits (99.1%) and of greeneries and vegetables (89, 3%) (Table 2). There was a significant association between high concentrations of LDL-cholesterol and lower fruit intake (Table 4). Hung et al. assessed the association between fruit intake and cardiovascular disease, fruits being protective when consumption was of ≥ 3 fruit servings per day [25].

**Table 4:** **Association between inadequate intake with clinical characteristics, inflammatory markers and food intake**

Characteristics	N (Total = 215)	Inadequate intake		
		Fruits (< 3 servings/day)	Vegetables and greenery (< 4 servings/day)	Sodium (> 2400 mg/day)
***Outpatient clinics***				
Hormone therapy				
p-value*		0.26	0.34	**0.02**
Yes	30 (14.0%)	29 (96.7%)	28 (93.3%)	11 (36.7%)
No	185 (86.0%)	184 (99.5%)	164 (88.6%)	108 (58.4%)
***Inflammatory markers***				
Glucose levels (mg/dL)				
p-value		0.39	0.07	0.41
<100	80 (37.2%)	80 (100.0%)	75 (93.8%)	43 (53.8%)
≥100	135 (62.8%)	133 (98.5%)	117 (86.7%)	76 (56.3%)
TC (mg/dl)				
p-value		0.07	0.47	**<0.001**
<200	60 (27.9%)	58 (96.7%)	53 (88.3%)	46 (76.7%)
≥200	155 (72.1%)	155 (100.0%)	139 (89.7%)	73 (47.1%)
LDLC (mg/dl)				
p-value		**0.03**	0.56	**0.02**
<100	40 (18.6%)	38 (95.0%)	36 (90.0%)	28 (70.0%)
≥100	175 (81.4%)	175 (100.0%)	156 (89.1%)	91 (52.0%)
HDLC (mg/dl)				
p-value		0.42	0.59	0.5
<50	75 (34.9%)	75 (100.0%)	67 (89.3%)	33 (44.0%)
≥50	140 (65.1%)	138 (98.6%)	125 (89.3%)	63 (45.0%)
TG (mg/dl)				
p-value		0.65	0.52	0.09
<150	88 (40.9%)	87 (98.9%)	79 (89.8%)	54 (61.4%)
≥150	127 (59.1%)	126 (99.2%)	113 (89.0%)	65 (51.2%)
***Food intake***				
Total energy intake (kcal/day)				
p-value		0.55	0.09	**<0.001**
<2000	160 (74.4%)	158 (98.8%)	146 (91.3%)	68 (42.5%)
≥2000	55 (25.6%)	55 (100.0%)	46 (83.6%)	51 (92.7%)
Protein (%)				
p-value		1	0.72	0.06
<10	7 (3.3%)	7 (100.0%)	6 (85.7%)	1 (14.3%)
10–35	204 (94.9%)	202 (99.0%)	182 (89.2%)	115 (56.4%)
>35	4 (1.9%)	4 (100.0%)	4 (100.0%)	3 (75.0%)
Carbohydrate (%)				
p-value		**0.02**	0.18	0.05
<45	77 (35.8%)	75 (97.4%)	72 (93.5%)	48 (62.3%)
45–65	129 (60.0%)	129 (100.0%)	111 (86.0%)	69 (53.5%)
>65	9 (4.2%)	9 (100.0%)	9 (100.0%)	2 (22.2%)
Lipids (%)				
p-value		0.53	0.29	**0.01**
<20	7 (3.3%)	7 (100.0%)	7 (100.0%)	1 (14.3%)
20–30	94 (43.7%)	94 (100.0%)	87 (92.6%)	47 (50.0%)
>30	114 (53.0%)	112 (98.2%)	98 (86.0%)	71 (62.3%)
Saturated Fat (%)				
p-value		0.73	0.06	0.08
<10	111 (51.6%)	110 (99.1%)	103 (92.8%)	56 (50.5%)
≥10	104 (48.4%)	103 (99.0%)	89 (85.6%)	63 (60.6%)
Cholesterol (mg)				
p-value		0.06	**<0.001**	**<0.001**
≥300	168 (78.1%)	166 (98.8%)	161 (95.8%)	83 (49.4%)
>300	47 (21.9%)	47 (100.0%)	31 (66.0%)	36 (76.6%)

Data are expressed in numbers and percentage between parentheses.

*statistical difference between groups p <0.05 (Fisher’s Exact Test).

Another study found that low intake of fruits represented both, overall risk for obesity and for abdominal fat Perozzo et al. [26] studied the association between food patterns and obesity in a population based cross sectional study, with a representative sample of 1.026 women (20–60 years) in São Leopoldo, Rio Grande do Sul, Brazil. Overall and abdominal obesity were present in 18% and 23.3% women respectively. After controlling for confounding factors, low fruit intake was positively associated with BMI [26]. The present study did not find such an association.

Identifying the type of body fat distribution is crucial because the accumulation of fat in the abdominal region is directly linked to metabolic changes that can lead to the development of cardiovascular diseases and diabetes mellitus. During the menopause, the decrease in estrogen and the overall increase of body weight are concurrent with the augmentation of visceral fat (abdominal). This characterizes an android profile associated with higher cardiovascular risks in postmenopausal women [27]. Toth et al. reported a 49% increase in abdominal fat and 22% of subcutaneous fat in postmenopausal women compared to women between the first and last natural menstruation [2].

In this study, the prevalence of high WC as a cardiometabolic risk factor was high (92.1%), as well as glucose levels (62.8%) and triglycerides (59.1%) (Table 2). Cardiometabolic risk factors and diet quality are not significantly associated in this study (Table 2), corroborating the findings reported by Tardivo et al. [21]. These authors also found an association between diet quality and total body fat estimated by skinfold, a measurement not collected measurement in the present study. Nonetheless, this study showed that women under 65 and overweight have a higher prevalence of hypertension and hyperglycemia and that the excessive intra-abdominal fat relates to glucose intolerance and insulin resistance (Table 5).

**Table 5:** **Association between cardiometabolic risk factors and clinical characteristics**

Characteristics	N (Total = 215)	Increased WC (≥80 cm)	Low HDL (<50 mg/dl)	Increased TG (≥150 mg/dl)	High blood pressure (≥130 × 85 mmHg)	Increased glucose levels (≥100 mg/dl)
***Outpatient clinics***						
BMI <65 years old						
p-value*		**<0.001**	0.25	0.42	**0.03**	**0.01**
Eutrophic	48 (28.7%)	36 (75.0%)	19 (39.6%)	25 (52.1%)	9 (18.8%)	22 (45.8%)
Overweight	63 (37.7%)	62 (98.4%)	19 (30.2%)	39 (61.9%)	21 (33.3%)	44 (69.8%)
Obese	56 (33.6%)	56 (100.0%)	25 (44.6%)	36 (64.3%)	24 (42.9%)	39 (69.6%)
BMI ≥ 65 years old						
p-value*		**<0.001**	0.12	0.47	0.71	0.24
Lean	5 (10.4%)	3 (60.0%)	1 (20.0%)	3 (60.0%)	1 (20.0%)	4 (80.0%)
Eutrophic	14 (29.2%)	12 (85.7%)	1 (7.1%)	6 (42.9%)	3 (21.4%)	6 (42.9%)
Overweight	29 (60.4%)	29 (100.0%)	10 (34.5%)	18 (62.1%)	10 (34.5%)	20 (69.0%)
***Lifestyle***						
Self-reported lifestyle						
p-value*		0.51	0.24	0.09	0.49	0.39
Active	71 (33.0%)	65 (91.5%)	22 (31.0%)	37 (52.1%)	23 (32.4%)	46 (64.8%)
Sedentary	144 (67.0%)	133 (92.4%)	53 (36.8%)	90 (62.5%)	45 (31.3%)	89 (61.8%)
Aerobic exercises						
p-value*		0.64	0.2	0.48	0.06	0.52
Yes	14 (6.5%)	11 (78.6%)	2 (14.3%)	7 (50.0%)	2 (14.3%)	10 (71.4%)
No	25 (11.6%)	22 (88.0%)	8 (32.0%)	14 (56.0%)	11 (44.0%)	19 (76.0%)
Strengthening exercises						
p-value*		0.16	0.2	0.12	0.45	0.38
Yes	5 (2.3%)	3 (60.0%)	0 (0.0%)	1 (20.0%)	1 (20.0%)	3 (60.0%)
No	34 (15.8%)	30 (88.2%)	10 (29.4%)	20 (58.8%)	12 (35.3%)	26 (76.5%)

Data are expressed in numbers and percentage between parentheses.

*statistical difference between groups p <0.05 (Fisher’s Exact Test).

The high WC as surrogate measure for the accumulation of intra-abdominal fat, is directly associated with the prevalence of diabetes, increasing the risk of cardiovascular disease. Before menopause, women have lower levels of blood pressure than men of the same age group do. After menopause, the blood pressure levels of women exceed those of men in the same age range. Hypoestrogenism during the postmenopause causes a tendency to increase blood pressure thus increasing the risk of cardiovascular diseases [1].

The mean daily sodium intake was of 2829.9 mg (Table 1), is above the maximum recommended intake for adults (2400 mg) [14] and less than that reported by the Research Project on Household Budgets (POF) 2008–2009 (12 g of salt or 4700 mg of sodium) [22]. Women (7%) also reported adding salt to prepared meals. Being aware that this is quite a popular practice, we may consider this figure underrated. Martinazzo et al. reported a prevalence of 16.7% of excessive sodium intake, which is roughly 2.4 fold higher than the level found in this study [28]. However, this level of excessive sodium intake was associated with higher serum concentrations of total cholesterol and LDLC, increased lipids and dietary cholesterol, and even lack of hormone replacement therapy (Table 4). The profile of inadequate dietary intake of sodium and lipids is associated with lower levels of estrogens and favors atherogenesis. Along the aging process, women experience variations in their metabolic profile leading to changes in the composition and distribution of adipose tissue, favoring not only weight gain, but also the progression of atherosclerotic processes. Estrogen deficiency is not the only predisposing factor for weight gain after menopause. It is often parallel to lower basal metabolic rate and a tendency to adopt a more sedentary lifestyle, subsequent to the aging process [29].

The risk of developing coronary artery disease related to sedentary life is 1.5 to 2.4 times higher when compared to hypertension, dyslipidemia and smoking. Aerobic physical activity of moderate intensity when performed on a regular basis (at least 30 minutes, three times a week), can have an impact in reducing the risk of cardiovascular events in the range 30–40% [11]. In this study, although 33% of women are self-reported as active (Table 2) and 74.4% consumed a normal caloric diet (less than 2000 kcal/day), there is a high prevalence of overweight and obesity as well as high WC. Only 8.8% actually performed aerobic or strengthening exercises sufficiently enough to have an impact on the prevention of cardiovascular diseases [12]. Women who do not perform physical activities were more likely to have high blood pressure (44%) when compared to those who were active (14.3%) (Table 5). Regular physical activities is an important therapeutic, non-pharmacological preventive method against cardiovascular events.

The following study limitations are important when interpreting the findings reported herein. First, the sample size was relatively small due to the type of the study design (cross-sectional study, based on a convenience sample). Thus, the study results do not reflect either the nutritional status, or the health patterns of the population of Rio de Janeiro. Second, the variation of food consumption exists between individuals (inter-individual variability) and in the same individual, in relation to daily intakes (intra-individual variability), and these are inherent to studies of this type. Moreover, estimated food consumption methods are marked by variations along the evaluation process, from obtaining individuals reported information to the compilation of data. These could lead to a misleading use of data related to food consumption patterns and their association with health outcomes. Third, although the 24-hour recall method is widely used for dietary assessments, the intake of a single day does not represent the daily intake of an individual. However, it has been carefully considered to be the most appropriate and feasible tool regarding both, the purpose of this study and the study population. According to Willett, applying a single 24-hour recall may be suitable for estimating mean intakes in groups, given a sample size that suits this purpose [30].

## Conclusions

This study concludes that the women consumed a low quality diet attributed to the low intake of fruits, vegetables and greeneries and excessive sodium. These inappropriate eating practices have a negative impact on cardiometabolic risk factors of postmenopausal women who also showed a high prevalence of abdominal obesity. Furthermore, women presented increased lipids, fasting glucose as well as higher blood pressure levels that are recognized markers of increased cardiovascular risks.

## Authors’ information

DAV is a Nutritionist and has a MSc Degree (Instituto Fernandes Figueira–IFF/FIOCRUZ); VMF has a PhD in Epidemiology and works in Clinical Research. EGR has a PhD in Biomedical Engineering and works in Clinical Research; LPFM has a PhD in Child and Women’s Heath and heads the Endocrinology Department; RAGS has a PhD in Collective Health; CRMMC has a PhD in Internal Medicine and works at Department of Food and Nutrition. MVMP has a PhD in Biomedical Engineering and works in Clinical Research.
